# Organ-Specific Mitochondrial Alterations Following Ischemia–Reperfusion Injury in Post-Cardiac Arrest Syndrome: A Comprehensive Review

**DOI:** 10.3390/life14040477

**Published:** 2024-04-05

**Authors:** Eriko Nakamura, Tomoaki Aoki, Yusuke Endo, Jacob Kazmi, Jun Hagiwara, Cyrus E. Kuschner, Tai Yin, Junhwan Kim, Lance B. Becker, Kei Hayashida

**Affiliations:** 1 Laboratory for Critical Care Physiology, Feinstein Institutes for Medical Research, Northwell Health System, Manhasset, NY 11030, USA; eriko.nakamura@ompu.ac.jp (E.N.); taoki@northwell.edu (T.A.); yendo@northwell.edu (Y.E.); jkazmi@northwell.edu (J.K.); jhagiwara@northwell.edu (J.H.); ckuschner1@northwell.edu (C.E.K.); tyin@northwell.edu (T.Y.); jkim46@northwell.edu (J.K.); lance.becker@northwell.edu (L.B.B.); 2 Department of Emergency Medicine, Donald and Barbara Zucker School of Medicine at Hofstra/Northwell, Hempstead, NY 11549, USA

**Keywords:** mitochondria, ischemia, reperfusion injury, cardiac arrest, ischemic stroke, cardiac injury, acute kidney injury

## Abstract

Background: Mitochondrial dysfunction, which is triggered by systemic ischemia–reperfusion (IR) injury and affects various organs, is a key factor in the development of post-cardiac arrest syndrome (PCAS). Current research on PCAS primarily addresses generalized mitochondrial responses, resulting in a knowledge gap regarding organ-specific mitochondrial dynamics. This review focuses on the organ-specific mitochondrial responses to IR injury, particularly examining the brain, heart, and kidneys, to highlight potential therapeutic strategies targeting mitochondrial dysfunction to enhance outcomes post-IR injury. Methods and Results: We conducted a narrative review examining recent advancements in mitochondrial research related to IR injury. Mitochondrial responses to IR injury exhibit considerable variation across different organ systems, influenced by unique mitochondrial structures, bioenergetics, and antioxidative capacities. Each organ demonstrates distinct mitochondrial behaviors that have evolved to fulfill specific metabolic and functional needs. For example, cerebral mitochondria display dynamic responses that can be both protective and detrimental to neuronal activity and function during ischemic events. Cardiac mitochondria show vulnerability to IR-induced oxidative stress, while renal mitochondria exhibit a unique pattern of fission and fusion, closely linked to their susceptibility to acute kidney injury. This organ-specific heterogeneity in mitochondrial responses requires the development of tailored interventions. Progress in mitochondrial medicine, especially in the realms of genomics and metabolomics, is paving the way for innovative strategies to combat mitochondrial dysfunction. Emerging techniques such as mitochondrial transplantation hold the potential to revolutionize the management of IR injury in resuscitation science. Conclusions: The investigation into organ-specific mitochondrial responses to IR injury is pivotal in the realm of resuscitation research, particularly within the context of PCAS. This nuanced understanding holds the promise of revolutionizing PCAS management, addressing the unique mitochondrial dysfunctions observed in critical organs affected by IR injury.

## 1. Introduction

Post-cardiac arrest syndrome (PCAS) involves a complex constellation of pathophysiological processes triggered by the restoration of systemic circulation after successful cardiopulmonary resuscitation (CPR). Characterized by cerebral injury, myocardial dysfunction, systemic ischemia–reperfusion (IR) response, and underlying pathologies, this syndrome contributes significantly to morbidity and mortality in survivors of cardiac arrest [1,2]. At its core, mitochondrial dysfunction plays a pivotal role, being both affected by and contributing to IR injury during PCAS [3,4,5,6,7,8]. In this context, targeting organ-specific mitochondrial dysfunction represents a novel therapeutic approach but remains minimally explored. For instance, the brain and heart, being highly aerobic organs, might demonstrate distinct mitochondrial adaptive or maladaptive changes post-resuscitation, which could influence the overall outcome of PCAS. Therefore, investigating these organ-specific mitochondrial alterations can also facilitate the development of targeted interventions aimed at mitigating mitochondrial dysfunction and enhancing patient recovery from PCAS. This nuanced approach necessitates a paradigm shift in current research methodologies, advocating for a more granular exploration of mitochondrial function across different organs to further elucidate the pathophysiology of PCAS and unlock new avenues for patient-specific therapeutic strategies.

IR injury occurs in scenarios such as cardiac arrest, myocardial infarctions, cerebrovascular events, and organ transplantation [9,10,11,12,13,14]. IR injury involves a cascade of biochemical and cellular events leading to significant tissue damage, contributing to morbidity and mortality [13,15,16,17]. While the exact mechanisms of IR injury are not fully understood, mitochondrial dysfunction is believed to play a significant role [18,19,20,21,22].

Current research on PCAS primarily addresses generalized mitochondrial responses, resulting in a knowledge gap regarding organ-specific mitochondrial dynamics. Mitochondrial responses to IR vary across organ systems, influenced by differences in mitochondrial structure, bioenergetics, antioxidative capacities, and ROS-mediated damage susceptibilities. Thus, understanding organ-specific responses will allow researchers to understand the pathophysiology of IR injury and develop targeted therapeutics in the context of PCAS. This review summarizes the current understanding of mitochondrial roles in IR injuries across brain, heart, and kidney tissue. We also explore potential therapeutic strategies targeting mitochondrial dysfunction to enhance outcomes in post-IR.

## 2. The Pivotal Role of Mitochondria in Cellular Bioenergetics and Homeostasis

Mitochondria are dynamic, multifaceted organelles that play a pivotal role in cellular bioenergetics, metabolism, and signaling [23,24,25,26,27,28]. Their complex dual-membrane architecture is intricately designed to facilitate their diverse functions, and their physiology is tightly regulated to maintain cellular homeostasis. The outer membrane, acting as a protective barrier, is equipped with porins that facilitate the passage of ions and small molecules [29,30]. The inner mitochondrial membrane (IMM) is highly convoluted, containing cristae that increase the surface area, thereby enabling a wide range of biochemical processes. These cristae house the electron transport chain (ETC), a sophisticated assembly of proteins responsible for energy conversion [30,31,32]. The intermembrane space and the mitochondrial matrix each have their unique environments, adding to the organelle’s multifaceted functions. The IMM is particularly crucial for apoptosis, as it is involved in the release of caspase activators such as cytochrome *c* and changes in electron transport. Moreover, the IMM plays a role in the production of reactive oxygen species (ROS), which are implicated in various pathologies, such as cardiovascular, neurodegenerative, and inflammatory diseases [33,34,35].

Most importantly, mitochondria convert nutrients into adenosine triphosphate (ATP). This process involves a series of redox reactions in the ETC, where electrons are transferred across protein complexes, generating an electrochemical gradient known as the mitochondrial membrane potential (MMP). This potential is the driving force behind ATP synthesis through oxidative phosphorylation, a process vital for maintaining cellular homeostasis [29,30,31]. Beyond ATP production, mitochondria function as metabolic hubs, involved in fatty acid oxidation, the tricarboxylic acid cycle, and the urea cycle. Their roles extend to regulating calcium homeostasis, ROS modulation, and apoptosis, positioning them as key determinants of cell death [36,37,38,39].

Mitochondria do not serve an isolated function but rather are part of a synergistic interaction that influences various cellular processes. For instance, mitochondria work in synergy with organelles such as the endoplasmic reticulum and extracellular matrix to control the dynamic balance of the cell calcium concentration, critical for the physiological processes from muscle contraction to neurotransmitter release. They buffer intracellular calcium levels, thereby modulating a range of physiological processes from muscle contraction to neurotransmitter release [40,41,42]. Furthermore, mitochondria are dynamic organelles, constantly undergoing fusion and fission, processes that are not merely structural adjustments but are intrinsically tied to their function. Fusion allows for the mixing of mitochondrial contents, facilitating complementation and thus enhancing respiratory function. Conversely, fission enables the replication of mitochondria, as well as the isolation and removal of damaged mitochondrial components, serving as a quality control mechanism [43,44,45,46,47,48].

However, the high-energy demands of mitochondria make them susceptible to oxidative stress, particularly in metabolically active tissues. The very processes that generate ATP also produce ROS as by-products, creating a precarious balance between energy production and oxidative damage. When this balance is disrupted, mitochondria can activate apoptotic pathways, triggering cell death. Yet, they also possess an innate resilience, with mechanisms like mitochondrial biogenesis and mitophagy serving as reparative strategies to restore function [49,50,51,52].

## 3. Organ-Specific Metabolism during Ischemia and Reperfusion

The vulnerability of specific tissues to IR injury is intricately linked to their inherent metabolic needs and mitochondrial resilience, leading to variable responses among vital organs, each with distinct physiological roles and metabolic requirements. We aim to underscore the critical need to identify these variations to develop precise interventions aimed at counteracting the adverse effects of IR injury by navigating the complex interrelation between metabolic anomalies and mitochondrial changes. Here, we review recent knowledge in understanding the organ-specific metabolic and mitochondrial alterations during IR injury.

### 3.1. Brain Metabolism and IR Injury

The cerebral metabolism under normoxic conditions predominantly harnesses glucose through aerobic pathways, ensuring efficient energy production for the brain’s high metabolic demands [53,54]. Ischemic events compel the brain to shift towards anaerobic glycolysis as an alternative energy source. This metabolic adaptation, while temporarily sustaining ATP production, results in the accumulation of lactate and a consequential reduction in intracellular pH [54,55]. This shift to anaerobic metabolism, though adaptive, sets the stage for significant cellular distress upon reperfusion [53,54]. The reintroduction of oxygen post-ischemia, rather than being wholly restorative, paradoxically exacerbates tissue damage through a phenomenon known as reperfusion injury. This is marked by an excessive generation of ROS, overwhelming the cellular antioxidant defenses and leading to oxidative stress, a key mediator of cellular injury and death [22]. The oxidative stress not only damages cellular proteins, lipids, and DNA but also further impairs mitochondrial function, creating a vicious cycle of mitochondrial damage and ROS production.

### 3.2. Heart Metabolism and IR Injury

The metabolic adaptability of the heart, characterized by its capacity to metabolize a range of substrates such as fatty acids, glucose, and lactate, is foundational to its robust functionality. This flexibility facilitates the heart’s ability to maintain ATP production under varying physiological conditions [56]. During ischemic events, however, this metabolic versatility is compromised due to restricted oxygen availability, leading to a decreased efficiency in ATP synthesis as the myocardium shifts from the predominant fatty acid oxidation to glycolysis, a less efficient energy production pathway in the context of oxygen scarcity. Reperfusion introduces additional challenges, notably mitochondrial calcium overload [57,58]. This influx of calcium into the mitochondria during reoxygenation exacerbates metabolic disarray and precipitates the opening of the mitochondrial permeability transition pore (mPTP), a critical event that disrupts mitochondrial integrity and contributes to cell death through necrosis and apoptosis [59]. This sequence of events underscores the complexity of mitochondrial dysfunction during IR injury, highlighting not just shifts in substrate preference but also the detrimental impact of impaired ETC activity, further compromising ATP generation and contributing to the energetic deficit within cardiac cells [60].

### 3.3. Kidney Metabolism and IR Injury

The kidneys demonstrate metabolic adaptability, with the cortical cells predominantly engaging in fatty acid oxidation, while the medullary cells rely more heavily on glycolysis for their energy needs. This metabolic specialization between the cortex and medulla not only reflects their different physiological roles but also suggests that they may have varying levels of vulnerability to IR injury [61]. The cortex, endowed with a high density of mitochondria, predominantly utilizes fatty acid oxidation, a process heavily reliant on oxygen, to efficiently produce ATP, supporting energy-intensive tasks such as filtration and active reabsorption. In contrast, the medulla, situated farther from the oxygen-rich blood supply and operating within the constraints of a hypoxic environment, primarily depends on glycolysis for energy [62]. This adaptation ensures functionality despite lower oxygen availability, albeit at the cost of reduced ATP yield per glucose molecule compared to oxidative phosphorylation. IR injury disrupts this delicate metabolic equilibrium, leading to significant alterations in energy dynamics [63].

## 4. Organ-Specific Mitochondrial Responses to Ischemia–Reperfusion Injury

### 4.1. Brain Mitochondrial Response and Vulnerability in IR Injury

Mitochondria play a crucial role in meeting the high energy demands of neurons [64]. The distribution and morphology of mitochondria vary significantly between axonal and dendritic compartments in neurons, reflecting their distinct functions and responses to neuronal activity [65]. Axonal mitochondria travel long distances along microtubules via forward molecular motors of the kinesin family and reverse molecular motors of the dynein family. Axonal mitochondria are smaller and more dynamic [66], while dendritic mitochondria are elongated and more stationary [67,68]. This differential regulation is key to maintaining neuronal integrity and function [66,69]. Neurons, especially in the human brain, require substantial amounts of ATP for various functions, including maintaining membrane potential and synaptic activity. While glycolysis was once thought to be the primary source of ATP in neurons [70], it is now understood that oxidative phosphorylation (OXPHOS) is the major energy source during neuronal activity [66,71]. Synaptic activity influences mitochondrial behavior and localization, with increased activity leading to changes in mitochondrial movement and positioning in both dendritic and axonal regions [67,71,72]. In the axon, increased neuronal activity results in a greater concentration of mitochondria at synaptic junctions and a reduction in their overall length [67,73]. On the other hand, previous studies showed that diminished synaptic activity is associated with a decrease in both the presence of mitochondria at presynaptic sites and the mobility of these mitochondria [67,73]. These adaptations are essential for fulfilling the substantial energy requirements of neuronal processes.

The brain’s mitochondrial landscape is unique, particularly in the context of IR injury [74,75,76,77]. Neuronal mitochondria, highly concentrated in neurites to fuel costly axonal transport, adopt an elongated and interconnected morphology, facilitating efficient energy distribution across extensive neuronal networks [78,79]. However, this specialization renders brain mitochondria susceptible to oxidative stress, a vulnerability exacerbated during IR injury [80]. The brain’s high oxygen consumption for ATP production via OXPHOS leads to an increased generation of ROS, which can overwhelm antioxidant defenses and induce oxidative stress. This stress, coupled with the susceptibility of the brain’s abundant polyunsaturated fatty acids to peroxidation, can damage mitochondrial membranes, affecting their structure and function. Consequently, these alterations can impair the ETC, reduce mitochondrial membrane potential, and decrease ATP synthesis, compromising neuronal function and health [81,82,83,84].

In addition to these structural and functional vulnerabilities, cerebral mitochondria are also influenced by specific regulatory mechanisms that affect their response to ischemic stress. Cerebral mitochondria are particularly susceptible to mitochondrial permeability transition (MPT), a process where the mPTP opens, allowing the passage of low molecular weight solutes across the typically impermeable inner mitochondrial membrane (IMM). This event can precipitate cell death by disrupting mitochondrial function. This is exacerbated by high levels of extracellular glutamate released during cerebral ischemia, which in turn triggers a large calcium influx and overwhelms mitochondrial regulation of calcium homeostasis [85,86,87]. The brain’s limited regenerative capacity compounds this heightened vulnerability, rendering neuronal cells particularly susceptible to irreversible damage [88,89]. Furthermore, the post-ischemic inflammatory response within the brain can exacerbate mitochondrial dysfunction, triggering a cascade of neurodegeneration and cognitive deficits [87,90]. This is thought to be mediated by the release of mitochondrial contents, such as mitochondrial DNA, cardiolipins, and ROS, which in turn activate the NLRP3 inflammasome in neighboring cells [87]. Cardiolipin, a crucial phospholipid of the IMM, is essential for the optimal function of cytochrome c oxidase and the stability of the ETC, directly impacting ATP synthesis and mitochondrial health. The NLRP3 inflammasome, activated by mitochondrial distress signals, orchestrates inflammatory responses, linking mitochondrial dysfunction to inflammation through the production of pro-inflammatory cytokines.

Cerebral IR can have profound consequences and often results from conditions such as stroke or cardiac arrest. In regional cerebral hypoxia, which is most often a result of ischemic stroke, astrocytic and neuroendothelial mitochondria can sense neurons’ metabolic hypoactivity and respond through the release of nitric oxide and other vasodilatory agents. This response can alter cerebral blood flow to augment collateral circulation in hypoperfused yet salvageable tissue and extend the critical window for recanalization [91,92]. In the case of PCAS, global ischemia affects the entire brain, contrasting with the localized brain damage seen in stroke. Even a brief period of global ischemia can inflict debilitating neurological damage as cerebral blood flow regulation is diminished [93]. Cerebral I/R injury disrupts the delicate balance between mitochondrial fusion and fission, critical processes for maintaining mitochondrial function and neuronal health. During ischemia, energy depletion leads to an increase in mitochondrial fission, resulting in fragmented mitochondria that are less efficient in ATP production. Upon reperfusion, the sudden influx of oxygen and substrates can exacerbate mitochondrial damage through excessive production of ROS, further promoting fission and inhibiting fusion, leading to neuronal dysfunction and cell death. Additionally, I/R injury can trigger the release of mitochondria or mitochondrial components from cells, a process that may contribute to inflammation and injury propagation by activating immune responses or by direct transfer of damaged mitochondria to neighboring cells, exacerbating the injury response [44,94,95,96,97]. These disturbances in mitochondrial dynamics result in excessive ROS production and neuronal apoptosis, ultimately leading to neuronal death.

Finally, current research suggests that mitochondria may be released into the extracellular environment and potentially transferred between cells [98,99]. In the context of brain injuries or diseases, these extracellular mitochondria may function as damage-associated molecular pattern molecules, assuming roles that are either beneficial or harmful, depending on the specific environmental conditions [98]. Understanding these brain-specific mitochondrial dynamics is crucial for developing targeted therapies to mitigate the devastating effects of IR injuries in the brain.

### 4.2. Heterogeneity of Cardiac Mitochondria and Their Role in IR Injury

The heart features a distinctive mitochondrial architecture. Cardiac mitochondria are not a homogenous population but rather exist in distinct subpopulations with unique structural and functional characteristics. These subpopulations, including subsarcolemmal mitochondria (SSM), interfibrillar mitochondria (IFM), and perinuclear mitochondria (PNM), are differentially impacted by various cardiac pathologies [100,101,102,103]. Cardiomyocytes contain high-density mitochondria, reflecting the heart’s significant energy requirements [104,105,106].

Understanding the structural and functional differences between SSM and IFM is crucial to understanding the mechanism of cardiac IR injury. The heart’s response to IR injury is intricately linked to the structural diversity of its mitochondria, which include SSM located beneath the cell membrane and IFM situated between myofibrils. SSM are primarily involved in generating ATP for basal cellular functions and are more susceptible to IR-induced oxidative stress due to their proximity to the cell membrane, where ionic imbalances and ROS production are more pronounced during reperfusion. IFM, on the other hand, are crucial for ATP production needed for contractile function and are more affected by changes in intracellular calcium levels during IR injury. This structural and functional heterogeneity means that different mitochondrial populations within cardiac cells may have varied thresholds for damage and repair in response to IR injury, influencing the heart’s overall resilience or susceptibility to such stress. IR injury contributes to mitochondrial alterations, along with other reperfusion-induced alterations, influencing cardiac contractile recovery and infarct size. The termination of OXPHOS leads to depolarization of mitochondrial membranes, ATP depletion, and inhibition of myocardial contractile function [107,108,109]. Ischemic injury progresses more swiftly in SSM, as evidenced in previous studies [110,111].

Research has shown that 45 min of ischemia impairs oxidative phosphorylation, specifically via the inhibition of cytochrome oxidase activity in SSM [110]. A study further revealed that ischemia specifically reduces cardiolipin in SSM, impacting oxidative phosphorylation through cytochrome oxidase. This effect is unique to SSM, as cardiolipin levels in IFM remain unaffected. The decrease in cardiolipin in SSM is directly associated with reduced oxidative phosphorylation, suggesting a link to the dysfunction in the ETC observed during myocardial ischemia [112]. Importantly, distinct populations of cardiac mitochondria may exhibit varying susceptibilities to ischemic damage, thereby shaping the overall impact of IR injury on myocardial function [109,112]. These distinctions could shed light on the varying responses of cardiac mitochondria to IR, potentially paving the way for novel therapeutic interventions aimed at preserving mitochondrial integrity and, subsequently, improving outcomes of myocardial disease.

### 4.3. Renal Mitochondrial Resilience and Vulnerability in Ischemia–Reperfusion Injury

The renal system depends on oxidative metabolism for bioenergetic demands. It thus possesses a distinctive mitochondrial architecture. This architecture is particularly evident in the mitochondria-dense regions of the proximal tubules and thick ascending limbs (TAL) [113,114,115]. Renal mitochondria play central roles in managing ROS, maintaining cellular calcium equilibrium, and modulating various signaling cascades. During IR injury, the protein dynamin-related protein 1 (Drp1) plays a crucial role in mitochondrial dynamics by mediating mitochondrial fission, which can lead to mitochondrial fragmentation and dysfunction. This process is associated with increased production of ROS and cellular damage, contributing to the pathophysiology of IR injury. The alteration in mitochondrial morphology and function following hypoxia is closely associated with the activation of Drp1 and its subsequent translocation to mitochondria [44,116]. This translocation is marked by enhanced Drp1-mediated mitochondrial fission and consequent apoptosis in tubular epithelial cells [117,118]. In this context, targeting Drp1 to modulate mitochondrial fission presents a potential therapeutic strategy to mitigate the detrimental effects of IR on mitochondrial and cellular health.

Despite renal metabolic plasticity facilitating adaptation to cellular stressors, this resilience is vulnerable to compromise under IR conditions, potentially culminating in acute kidney injury (AKI) [119,120,121,122]. Additionally, the kidneys receive about 20–25% of cardiac output, essential for maintaining adequate glomerular filtration, but exhibit heterogeneous blood flow and oxygen levels, particularly lower in the medulla. This heterogeneity, combined with varied metabolic activities of nephron segments, suggests that medullary mitochondria may have adapted to function efficiently in low-oxygen environments, supporting ATP production in suboptimal conditions [62].

Mitophagy, a selective process of degrading damaged mitochondria, has emerged as a critical player in the context of IR-induced AKI. This selective degradation mechanism ensures the removal of dysfunctional mitochondria, thereby preserving cellular integrity and function in the face of stress. The significance of mitophagy has been further elucidated through experimental studies involving knockout mice models deficient in BNIP3 (BCL2 interacting protein 3) and PINK1 (PTEN-induced kinase 1). BNIP3 and PINK1 are integral to the regulation of mitophagy, with BNIP3 directly facilitating the sequestration of damaged mitochondria and PINK1 initiating a cascade that tags these mitochondria for autophagic degradation [123,124]. Their coordinated action ensures the removal of dysfunctional mitochondria, thereby preserving cellular health and preventing mitochondrial-related pathologies. Research using knockout mice for BNIP3 and PINK1, two key regulators of mitophagy, has illuminated the protective effects of this process [125,126,127,128].

In summary, renal mitochondria are integral to preserving nephron integrity. Understanding the mitochondrial dynamics during renal IR injury is vital for innovating therapeutic interventions focused on maintaining mitochondrial health and attenuating the adverse impacts of IR on renal functionality [121,122,129].

## 5. Comparison of Mitochondrial Responses and Dynamics to Ischemia–Reperfusion Injury between the Brain, Heart, and Kidneys

**Figure 1:**
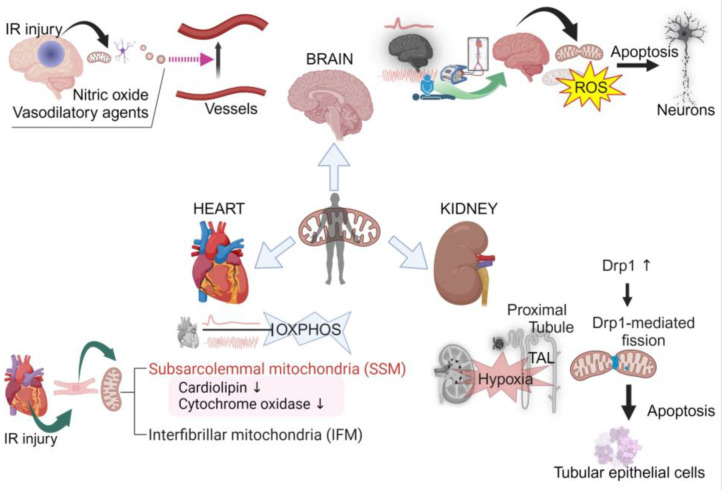
Comparison of mitochondrial responses to ischemia–reperfusion injury between the brain, heart, and kidneys. Created with BioRender.com.

As we have discussed, the brain, heart, and kidneys are particularly susceptible to IR injury. That susceptibility to injury is influenced by the structural and functional heterogeneity of mitochondria within these organs. Moreover, these organs exhibit diverse mitochondrial behaviors in the face of IR injury. The comparison of mitochondrial responses to IR injury between the brain, heart, and kidneys is shown in Figure 1.

In the brain, mitochondria, which are tailored for rapid energy distribution across neuronal networks, display heightened vulnerability to oxidative stress, exacerbated by the abundance of polyunsaturated fatty acids in cerebral tissue [74,75,76,77,80,81,82,83,84]. During cerebral ischemia, fusion is reduced, enhancing energy optimization, while reperfusion leads to increased fission and fragmentation, adapting to the influx of oxygen and nutrients but potentially generating ROS [22,96].

Conversely, myocardial mitochondria demonstrate a swift resurgence in oxidative phosphorylation upon reperfusion, with a temporary lag in cardiac contractility before restoring to pre-ischemic levels [107,108,109]. The cardiac tissue, highly reliant on fatty acid oxidation, shows vulnerability to perturbations in mitochondrial dynamics and oxidative stress [130,131,132]. The distinct susceptibilities of cardiac mitochondria to IR injury are compounded by their heterogeneous distribution into SSM and IFM populations [100,101,102,108,112,133].

In the kidneys, mitochondria predominantly populate the proximal tubules and TAL, adapting to reduced mass and structural alterations induced by ischemic insult [113,114,115,119,120,121,122,134]. Renal mitochondria exhibit a unique choreography of fission and fusion events, intrinsically tied to the organ’s susceptibility to acute injury [115,117,119].

To understand this organ-specific heterogeneity in mitochondrial dynamics, medical science must decipher the molecular mechanisms underlying them. This task requires an interdisciplinary approach encompassing cellular biology, biochemistry, and clinical medicine. The opportunity, however, is monumental: the potential to develop targeted therapeutic strategies that can mitigate the impact of IR injuries across a range of clinical scenarios, from cardiac arrest to organ transplantation.

## 6. Pharmaceutical Approaches in Ischemia–Reperfusion Injury with Targeted Mitochondrial Protection

A central element in the pathological process of IR injury is mitochondrial dysfunction. In response, strategies aimed at enhancing mitochondrial resilience and functionality have become pivotal in improving clinical outcomes. Pharmacological interventions that modulate mitochondrial function during IR injury show promise, especially those stabilizing mitochondrial processes and inhibiting mPTP formation. The application of mitochondria-targeted protective agents, including specific drugs and antioxidants, has shown encouraging effects in various in vivo models. For instance, Cyclosporine A (CsA) exerts a protective effect in myocardial ischemia by specifically targeting the mPTP. CsA binds to cyclophilin D, a key regulatory component of the mPTP located within the IMM, inhibiting the mPTP opening. This interaction is crucial for maintaining mitochondrial integrity, preventing calcium overload-induced mitochondrial swelling, and thus reducing cell necrosis post-myocardial ischemia. Furthermore, the ability of CsA to enhance mitochondrial calcium buffering capacity, as demonstrated in cardiac-isolated mitochondria, contributes to its protective role by delaying mPTP opening and preserving mitochondrial function [135,136,137,138,139]. However, the clinical application of Cyclosporine A in treating IR injury remains uncertain due to potential adverse effects and inconclusive benefits in clinical studies to date [140]. The mitochondria-targeted peptide SS-31, also known as elamipretide or Bendavia, concentrates within the IMM through interaction with cardiolipin, enhancing its activity. SS-31 offers a multifaceted protective role against IR injury by preserving mitochondrial function, reducing oxidative stress and inflammation, inhibiting MPT, protecting cellular integrity, and promoting tissue regeneration. By binding to cardiolipin, SS-31 stabilizes the IMM structure and supports the organization of respiratory complexes into supercomplexes, thereby enhancing OXPHOS and ATP production and decreasing the production of ROS. SS-31 also prevents mPTP opening, thereby maintaining MMP and preventing the release of pro-apoptotic factors. These actions collectively contribute to mitigating the deleterious effects of IR injury on affected tissues, particularly the kidneys [141,142]. Poloxamer-188 (P-188) exerts its protective effects against IR injury primarily by preserving the integrity of cell membranes and BBB. It achieves this by sealing defects in cell membranes, as demonstrated by reduced uptake of propidium iodide, indicating decreased cell membrane permeability. Additionally, P-188 inhibits the activation of matrix metalloproteinase-9, which is involved in extracellular matrix degradation and contributes to BBB disruption. By maintaining cellular and vascular integrity, P-188 mitigates the detrimental effects of IR injury [143,144,145].

## 7. Mitochondrial Transplantation as an Emerging Strategy for IR Injury

Mitochondrial transplantation (MTx) has emerged as a groundbreaking concept in IR injury [146,147]. Recent evidence suggests the potential for mitochondria to be released and transferred between cells. Notably, studies have demonstrated the transfer of mitochondria from astrocytes and microglia to damaged neurons, conferring neuroprotection [99,148]. Treatments with isolated exogenous mitochondria have significantly reduced infarct size in post-stroke rat models [149], and transplantation of muscle-derived mitochondria has been shown to alleviate cellular oxidative stress and apoptosis, decrease brain infarct volume, and reverse neurological deficits following ischemic stroke in rats [150]. In lung injury scenarios, bone marrow-derived stromal cells have been observed to release mitochondria, which are then transferred into pulmonary alveoli to mitigate damage [151]. While MTx as a treatment modality should work in brain, heart, and kidney tissue, work remains to be conducted to determine if each organ requires a different dose or delivery mechanism for MTx to be most effective.

In a rat model of cardiac arrest, the intravenous injection of freshly prepared donor mitochondria post-cardiac arrest and resuscitation significantly enhanced lung function, improved brain microcirculation, accelerated lactate clearance, and markedly increased 72 h survival rates [152]. Labeled donor mitochondria were detected in the brain and other organs 24 h post-cardiac arrest, substantiating the spontaneous transfer of exogenous mitochondria into neurons in vitro and highlighting the importance of mitochondrial membrane integrity for successful in vivo MTx. The retention of donor mitochondria in the brain following MTx potentially contributes to improved survival rates post-cardiac arrest [152]. Emerging research suggests that mitochondria are highly mobile, frequently traversing between cells, indicating that MTx could be beneficial for various organs.

A significant hurdle in MTx is the effective integration of exogenous mitochondria into recipient cells. This integration is complicated by the dynamic nature of mitochondrial networks within cells, which undergo continuous fusion and fission events. These dynamics are vital for maintaining mitochondrial function and adapting to fluctuations in cellular energy demands. Achieving successful integration of transplanted mitochondria into these networks is crucial for realizing their therapeutic potential.

One major concern of MTx is the potential immunogenicity of the transplanted mitochondria, which could trigger rejection or inflammatory responses in the recipient. Moreover, there is a risk associated with transferring damaged or dysfunctional mitochondria, potentially worsening existing conditions or introducing new cellular dysfunctions. The efficacy of MTx also could rely on the development of precise delivery methods to ensure that mitochondria are accurately targeted to the tissues or cells of interest, a task made challenging by the need to navigate biological barriers and minimize off-target effects.

Despite these challenges and limitations, MTx holds significant promise for treating not only IR injury-related diseases but also a broad spectrum of diseases linked to mitochondrial dysfunction. For instance, in neurodegenerative diseases where mitochondrial dysfunction is a contributing factor, such as Parkinson’s and Alzheimer’s, MTx could offer a novel therapeutic pathway.

## 8. Future Directions

Targeting organ-specific mitochondrial responses to whole-body IR injury presents a highly promising strategy for treating PCAS. Tailored strategies that include antioxidants and MPT inhibitors for neuroprotection, interventions to enhance oxidative phosphorylation in the heart, and methods to preserve mitochondrial mass in the kidneys are crucial. Additionally, the integration of next-generation sequencing and metabolomics has revealed the genetic and metabolic factors underlying mitochondrial dysfunction, guiding the development of targeted therapies [153,154,155]. Exploring epigenetic modulators, especially histone deacetylase inhibitors, offers new possibilities for regulating mitochondrial biogenesis and function, presenting innovative ways to modulate cellular energy dynamics [156,157]. This shift towards a patient-centric model marks a significant change in ischemia–reperfusion injury management, promising more nuanced and effective treatment strategies.

## 9. Conclusions

The elucidation of organ-specific mitochondrial alterations in response to IR injury in the context of PCAS underscores the imperative for tailored therapeutic interventions. The divergent mitochondrial dynamics observed in the brain, heart, and kidneys necessitate a nuanced approach to treatment, one that directly addresses the unique mitochondrial susceptibilities and adaptive mechanisms within these organs. The advent of mitochondrial medicine, bolstered by genomic and metabolomic innovations, is paving the way for more precise, condition-specific therapeutic strategies. Furthermore, the promising horizon of MTx offers a novel therapeutic modality, potentially revolutionizing the management of IR injury. These advancements not only promise to ameliorate the detrimental effects of IR injury but also herald a transformative era in critical care medicine, where treatments are increasingly personalized, targeting the mitochondrial epicenter of cellular dysfunction.

## Abbreviations

IR:	ischemia–reperfusion
IMM:	inner mitochondrial membrane
ETC:	electron transport chain
ROS:	reactive oxygen species
ATP:	adenosine triphosphate
MMP:	mitochondrial membrane potential
mPTP:	mitochondrial permeability transition pore
OXPHOS:	oxidative phosphorylation
MPT:	mitochondrial permeability transition
SSM:	subsarcolemmal mitochondria
IFM:	interfibrillar mitochondria
PNM:	perinuclear mitochondria
TAL:	thick ascending limbs
Drp1:	dynamin-related protein 1
AKI:	acute kidney injury
BNIP3:	BCL2/adenovirus E1B 19kDa interacting protein 3
PINK1:	PTEN-induced putative kinase 1
MTx:	mitochondrial transplantation
